# What factors affect clinical decision-making about access to stroke rehabilitation? A systematic review

**DOI:** 10.1177/0269215518808000

**Published:** 2018-10-29

**Authors:** Verity Longley, Sarah Peters, Caroline Swarbrick, Audrey Bowen

**Affiliations:** MAHSC and The University of Manchester, Manchester, UK

**Keywords:** Stroke, rehabilitation, decision-making, clinical reasoning, access

## Abstract

**Objectives:**

To determine the factors affecting clinical decision-making about which patients should receive stroke rehabilitation.

**Methods:**

Data sources (MEDLINE, CINAHL, AMED and PsycINFO) were searched systematically from database inception to August 2018. Full-text English-language studies of data from stroke clinicians were included. Studies of patients were excluded. The included studies were any design focussed on clinical decision-making for referral or admission into stroke rehabilitation. Summary factors were compiled from each included study. The quality of the included studies was assessed using the Mixed Methods Appraisal Tool.

**Results:**

After removing duplicates, 1915 papers were identified, of which 13 met the inclusion criteria. Eight included studies were qualitative and one used mixed methods. A total of 292 clinicians were included in the studies. Quality of the included studies was mixed. Patient-level and organizational factors as well as characteristics of individual clinicians contributed to decisions about rehabilitation. The most often described factors were patients’ pre- and poststroke function (*n* = 6 studies), presence of dementia (*n* = 6), patients’ social/family support (*n* = 6), organizational service pressures (*n* = 7) and the decision-making clinician’s own knowledge (*n* = 5) and emotions (*n* = 5).

**Conclusion:**

The results highlight a lack of clinical guidance to aid decision-making and reveal that a subjective approach to rehabilitation decision-making influenced by patient-level and organizational factors alongside clinicians’ characteristics occurs across services and countries.

## Introduction

Although coordinated multidisciplinary rehabilitation for patients following stroke improves mortality and independence, not every patient is selected to receive this intervention even though there is no evidence to indicate that certain patients will or will not benefit from rehabilitation.^1,2^ The benefits of stroke rehabilitation have been found in patients regardless of gender, age, stroke type and severity;^2^ however, internationally, there is disparity as to who does, or does not, receive stroke rehabilitation.^3^ Exclusions to rehabilitation services vary across current international clinical guidelines; in Canada, patients must demonstrate the potential ability to return to prestroke levels of function or to increase poststroke functional level;^4^ in the United States, patients must aim to be discharged into the community in order to receive inpatient rehabilitation.^5^ In Spanish guidelines, rehabilitation is not recommended for patients with severe stroke and “poor recovery prognosis.”^3^ Conversely, many clinical guidelines do not define which patients *should* receive stroke rehabilitation.^6^ The most recent UK clinical guidelines do not specifically exclude any types of patients; however, there are no criteria for who should access rehabilitation either.^3,7^ The decision of who should receive stroke rehabilitation therefore requires complex deliberation on the part of clinicians.

An increasing body of literature examines how clinicians choose which patients to refer or admit for stroke rehabilitation; however, this often focuses on patient factors and prognostic indicators, rather than investigating the clinician’s role in decision-making.^6,8,9^ The most recent systematic review of this topic is over seven years old and only used patient studies.^8^ Qualitative investigation reveals that decision-making about rehabilitation is a complex process requiring clinicians’ interpretation of clinical and non-clinical factors.^10^ Synthesizing the current literature on clinicians’ perspectives will help inform clinicians’ own decision-making process and also understand biases that may lead to inequalities in access.^11^ The aim of this review is to identify factors that affect clinical decision-making about who should receive stroke rehabilitation.

## Methods

Searches were completed on four electronic databases that focus on medical, allied health and psychology journals (all from inception to August 2018): Cumulative Index to Nursing and Allied Health Literature (CINAHL; via EBSCO search platform), PsycINFO (via Ovid), MEDLINE (via EBSCO) and Allied and Complementary Medicine (AMED; via Ovid). In addition, the Cochrane Central Register of Controlled Trials (CENTRAL; Issue 8, 2018) and Cochrane Stroke Group Trials Register (August 2018) were also searched. No restrictions were placed on study design or publication date, with English language being the only restriction. The search terms were adapted to terminology used by each database (see Appendix 1 for an example of the search strategy).

Studies were eligible for inclusion if they were full-text primary research published in peer-reviewed journals in which participants provided any type of stroke service (i.e. acute, rehabilitation or community). Studies were included that focussed on clinical decision-making for referral/admission to stroke rehabilitation, or that examined clinicians’ prioritization criteria for stroke rehabilitation or decision-making about rehabilitation potential, in essence any type of decision-making influencing subsequent access to stroke rehabilitation services. Studies focussed on decision-making between specific interventions or treatments were excluded, for example, decisions about which patients should receive a home visit.^12^ Studies that included a mixed diagnosis caseload (i.e. participants working in generic services) were excluded unless separate results for stroke were reported. Studies with patient participants were excluded. No restrictions were placed on the included study design.

The first author (V.L.) reviewed studies against the inclusion criteria by title. All abstracts and full text were then reviewed for eligibility by V.L. and a reviewer independent of the research team in order to minimize selection bias. Two discrepancies in inclusion were resolved through discussion. Reference lists of the included studies and relevant review papers were hand-searched for studies not already identified in the searches. We extracted all factors from the included studies and organized them into patient-level (e.g. patient’s age), organizational (e.g. staffing levels) and clinician-level (e.g. experience) factors.

In order to appraise the quality of studies, the Mixed Methods Appraisal Tool^13^ was used. This allows for the appraisal of qualitative and quantitative study designs simultaneously and scores studies on design, sampling, appropriateness of outcome measures and analysis method, randomization (when appropriate) and completeness of data. Studies are scored in four domains and the total scores ranged from 0% to 100%. All studies were assessed by V.L. and an independent reviewer, and the scores were compared. Two discrepancies were resolved through discussion. Studies were included regardless of quality rating.

## Results

**Figure 1.**
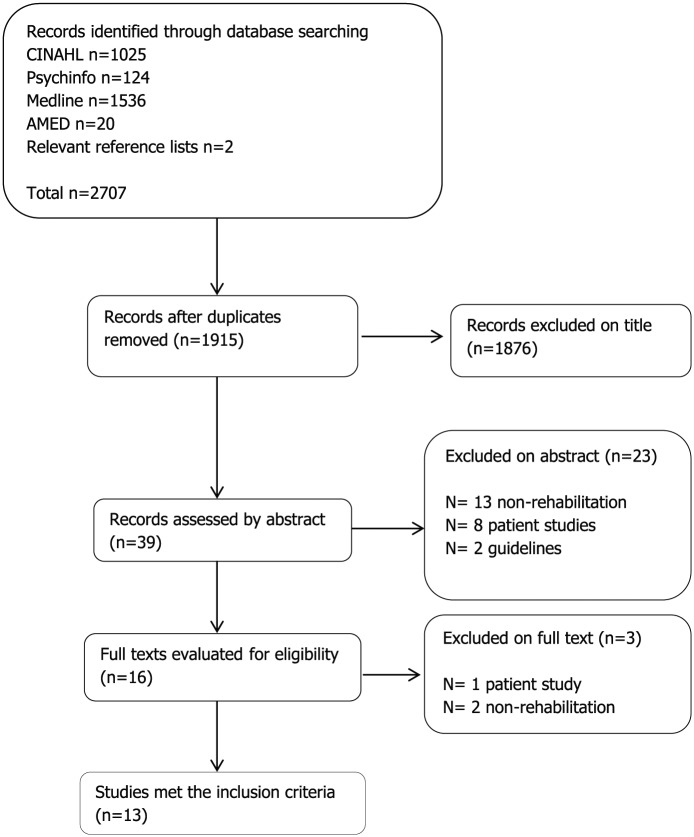
Flowchart of the literature searches.

After removing duplicates, 1915 papers were identified, of which 13 met the inclusion criteria and were included (see Figure 1). Eight of these were qualitative and one was mixed methods. The remaining quantitative studies all used questionnaires/surveys (see Table 1).

**Table 1.** Summary of the included studies.

Author and country	Design	Aim	Setting	Sample	Key findings	Study quality appraisal score (%)
*Qualitative*						
Burton et al.,^14^ UK	Focus groups	To investigate the meaning and influence of rehabilitation potential on clinical practice	Two inpatient and five community rehabilitation teams	12 clinicians (occupational therapy (OT), physiotherapy, speech and language therapy, rehabilitation assistant)	Rehabilitation potential is predicted by the observation of carry-over and functional gain. Judgement of rehabilitation potential influenced by prioritizing workload, working around the system, balancing optimism and realism. Impacts on patients and staff	75
Daniëls et al.,^15^ the Netherlands and Belgium	Focus groups	Identifying occupational therapists (OTs) deliberations in stroke rehabilitation	12 rehabilitation units	13 OTs	Difficulties for OTs were around: focusing on adaption while the patient is focussed on recovery, being client centered and protective simultaneously, setting meaningful goals in an institutional environment	75
Johnson et al.,^16^ Canada	Ethnography	Examining factors influencing team decision-making when choosing poststroke discharge destination	One acute stroke unit	One team, 12 multidisciplinary team members (psychiatrist, speech therapist, OT, physiotherapy, nurses, social worker, discharge planner)	Decisions about discharge destination influenced by social, economic and policy factors, interactions between team members, condition of patient and social support	100
Lam Wai Shun et al.,^17^ Canada	Focus group	Identifying factors influencing OT’s perception of rehabilitation potential after stroke	Three acute and three rehabilitation hospitals	12 OTs	Agreed on 11 most important patient-related factors to consider when assessing rehabilitation potential, and additional factors of the organizational context and clinician’s expertise	75
Longley et al.,^10^ UK	Semi-structured interview	To identify factors influencing clinicians’ decision-making about rehabilitation for people with preexisting cognitive impairment	Four inpatient and two community stroke teams	23 clinicians (OT, physiotherapy, speech and language therapy, psychology, nursing, physicians)	Decisions influenced by understanding of the individual patient, clinician’s knowledge of dementia/cognitive impairment, predicting rehabilitation potential, organizational constraints and clinician’s perceptions of their role within the team. Impacts on clinical practice	100
Luker et al.,^18^ Australia	Semi-structured interview	Exploring factors influencing Allied Health Professionals’ decision-making when prioritizing stroke rehabilitation	Three acute stroke units	15 Allied Health Professionals (physiotherapy, OT, speech and language therapy, dietician, social worker, psychologists)	Predicted discharge destination was a powerful driver of care decisions. Clinicians considered prestroke status, nature and severity of stroke, course of recovery and multiple factors within the healthcare system to aid decisions	75
Lynch et al.,^19^ Australia	Focus groups	Exploring factors perceived to affect why patients are referred to stroke rehabilitation and how assessments were completed	Eight acute stroke units	32 clinicians (mixed discipline)	Rehabilitation assessment and referral varied between units. People with stroke symptoms not consistently referred for rehabilitation. Perceived roles, beliefs about consequences, relationships with rehabilitation service providers and knowledge influenced decisions and referral practices	75
Lynch et al.,^20^ Australia	Observations and focus groups	Investigating how staff determine who to refer to rehabilitation	Eight acute stroke units	32 clinicians (nurse, physiotherapy, OT, speech therapy, dietician, unit manager). Meetings regarding 64 patients observed	Factors influencing referrals for rehabilitation: anticipated discharge destination, stroke severity, staff expectations, family advocacy; clinicians referred patients who they considered would be accepted	75
*Quantitative*						
Hakkennes et al.,^21^ Australia	Prospective observational cohort study, questionnaire	Identifying factors important in decision-making for rehabilitation	Five acute hospitals	14 rehabilitation assessors, 75 patients	Most important items for acceptance into rehabilitation: premorbid cognition, premorbid mobility, premorbid communication. For non-acceptance most important items: current mobility, social support, current cognition	100
Hasenbein et al.,^22^ Germany	Case-based survey	Analysis of medical decisions of allocation to stroke rehabilitation	Acute and rehab hospitals (unknown number)	33 physicians	Physician expertise and patient age influenced choice between in- or outpatient rehabilitation	50
Kennedy et al.,^23^ Australia	Questionnaire	Exploring factors influencing decisions for rehabilitation	12 rehabilitation units	17 physicians	Most influential clinical factors for accepting patients to rehabilitation: prognosis, social support, anticipated discharge destination, age, anticipated length of stay. Key non-clinical factors: priority, patient residence, workforce capacity	75
Magdon-Ismail et al.,^24^ USA	Survey	Investigating factors influencing selection of postacute discharge destination	471 acute hospitals	77 discharge planners	Factors influencing postdischarge care destination: insurance, quality of care facility, pressure to discharge patient. Patients and families more influential than physicians in choosing care facility. Non-clinical factors perceived to have major influence in decision-making	50
*Mixed methods*						
Putman et al.,^25^ Europe	Mixed methods: assessment, questionnaire, interview	Exploring factors involved in decision-making for admission to stroke rehabilitation	Six stroke rehabilitation units in four European countries (the United Kingdom, Belgium, Germany, Switzerland)	532 patients, medical consultants (unknown number)	Clinical criteria for admission evaluated differently between units: the United Kingdom only used diagnosis of stroke as admission criteria, Belgian, German and Swiss units all considered prestroke status and likelihood of discharge home in Swiss units	25

Study quality is scored and ranges from 0% to 100% according to % of criteria met in 25% intervals.^13^

Most included studies were conducted in Australia (*n* = 5).^18–21,23^ Two studies sampled from stroke units in more than one country.^15,25^ Three studies sampled clinicians involved in admitting patients to inpatient rehabilitation facilities and examined their admission criteria,^21,23,25^ and the others sampled referring clinicians. Two studies examined the factors influencing perceptions of rehabilitation potential after stroke, a complex concept sometimes used as a determinant for referring patients onto rehabilitation.^14,17^ A total of 292 clinicians were included in the studies. The study size varied from an ethnographic study of one multidisciplinary team^16^ to a survey of 77 discharge planners.^24^ And 671 patients were included across three of the studies,^20,21,25^ for example, in meetings observed about individual patients.

As shown in Table 1, the quality of studies was mixed. One mixed-methods study received a quality rating of 25%, three received a rating of 50%, seven received 75% and three met the full quality criteria of 100%. The eight qualitative studies were generally well conducted and reported, and all achieved a quality score of at least 75%. They needed greater clarity about their analysis process,^25^ how the findings may have related to researcher influence^14,17–20^ and how the findings may have related to context of the research.^15^ Three of the four quantitative studies had poor response rates (under 60%);^22,24,25^ some had an unrepresentative sample of the study population, for example, some participants were sampled due to participation in previous research^22^ or reasons for non-participation from eligible individuals were not explained,^23,24^ and there was a lack of clarity about the measures used.^25^

Patient-related factors (*n* = 8), organizational factors (*n* = 2) and the characteristics of individual clinicians (*n* = 4) were all found to influence clinicians’ decision-making for stroke rehabilitation. These key factors are described in Supplemental Table 1 and organized thematically below for clarity; however, categories were not mutually exclusive. Where possible, the positive or negative influences are described; however, some studies did not always specify the specific influence of factors. ^17,21,22,24^

### Patient-related factors

Five studies identified patient age as a factor perceived to influence decision-making (see Supplemental Table 1), three of which described older age as negatively affecting rehabilitation services received by patients.^16,18,22^ Older age was identified as a barrier for referral into some rehabilitation services although the reasons why were unexplored.^16^ Patient age was also used as a proxy for associated disability, for example, older people being assumed to have a lower baseline functioning.^18^

Pre- and poststroke functioning were factors both influencing decisions to refer and to admit (or decline) patients to rehabilitation. Putman et al.’s^25^ observational study of six stroke units across Europe found that higher levels of prestroke disability meant patients were less likely to be admitted. Similarly, Hakkennes et al.’s^21^ cohort study found that higher premorbid levels of function resulted in a higher likelihood of acceptance to rehabilitation, while poststroke factors were more important in not admitting patients. Lam Wai Shun et al.’s^17^ focus group study of occupational therapists found that pre- and poststroke function were 2 of 11 essential factors to consider when assessing a patient’s rehabilitation potential, although they did not state how this would affect decisions. Equally, pre- and poststroke status were considered important when prioritizing patients for rehabilitation by clinicians in Luker et al.’s^18^ interview study, especially when poststroke deficits such as swallowing difficulties increased patients’ risk of deterioration; higher priority was given to patients at risk. In addition, type and severity of stroke and interacting comorbidities were found to impact on decisions about rehabilitation, with patients with severe stroke being less likely to be referred for rehabilitation.^14,18,19^ Older patients with higher levels of prestroke disability or severe stroke appear less likely to be accepted or referred for rehabilitation.

A specific element of prestroke function, whether patients have preexisting dementia, was identified as an influential factor in decision-making not only affecting admission for rehabilitation, but also initial referrals. Lynch et al.^20^ found that clinicians were less likely to refer patients for rehabilitation when they believed that the patient would not be accepted, for example, patients with a diagnosis of dementia. Similarly, Lam Wai Shun et al.^17^ identified that patients with severe memory problems (although not specifically dementia) may be perceived to have low rehabilitation potential, as clinicians felt that they would be less likely to be accepted for rehabilitation. Neither study explored the reasons why staff believed this; however, in their other included study, Lynch et al.^19^ found that some participants considered that rehabilitation was not suitable for certain patients, particularly those with severe stroke or those with cognitive deficits/dementia. Clinicians perceived that these types of patients would never gain from rehabilitation, although the reasons why this was believed were not explored.^19^ Burton et al.^14^ found that some clinicians considered a premorbid diagnosis of dementia would indicate little rehabilitation potential and therefore limit the amount of rehabilitation that patients receive. Equally, clinicians in Longley et al.’s^10^ interview study found that clinicians perceived patients with dementia would lack rehabilitation potential or capacity to change unless they proved otherwise.

Putman et al.^25^ found in four out of six stroke rehabilitation units studied that premorbid cognitive disability reduced the likelihood of a decision to admit. One Belgian unit specifically screened patients for advanced dementia, although the study does not detail how the result of screening would influence admission and the methodological quality of this study was rated poorly when appraised. In addition, Hakkennes et al.^21^ surveyed assessors from rehabilitation units and viewed premorbid cognition as the most important item to consider when accepting patients for rehabilitation. It is not clear, however, whether the premorbid cognition specifically refers to dementia. The National Institutes of Health Stroke Scale (NIHSS)^26^ score was used to gather information about cognition which has no way of indicating a premorbid diagnosis of dementia. Again, it was not specified whether cognition would positively or negatively influence a decision for rehabilitation, just that it would be taken into account.

Social/family support was a factor that was considered to affect decisions about access to rehabilitation. A lack of social support, which may prevent patients from returning home, meant that patients were less likely to be admitted to one Swiss stroke unit in Putman et al.’s^25^ study when compared with other units in the study, although details remain unclear. Clinicians felt that families sometimes pressurized services into providing ongoing rehabilitation^18,25^ and influenced decisions about discharge destination.^24^ Home environment was also influential when deciding rehabilitation plans; patients from residential care were often not considered as candidates for rehabilitation unless families asked.^20^

Staff perceptions about patient-related factors also affected decision-making. Staff perception of patient motivation to engage in rehabilitation was found to affect decisions in five studies (see Supplemental Table 1). Occupational therapists in Daniëls et al.’s^15^ focus group study identified that patient motivation influenced their approach to rehabilitation and aided decisions on when to proceed with rehabilitation. While Daniëls et al.’s^15^ study focused on ongoing rather than access to rehabilitation, another study found that when participants were unmotivated to participate in therapy sessions, they were less likely to be referred for postacute rehabilitation in the first place.^18^ Two studies identified barriers to judging motivation, such as poststroke depression and attention,^14,18^ with some clinicians in Luker et al.’s^18^ acknowledging that low motivation would prevent access to rehabilitation but also feeling unable to influence it.

Patients were required to demonstrate progress with rehabilitation or have therapy-led goals in order to be referred for rehabilitation in five studies (see Supplemental Table 1). Observed improvement in the acute phase was an important factor in clinicians’ decision-making about whether a patient possessed rehabilitation potential.^17^ Lynch et al.^20^ found that a lack of improvement within the first two weeks post stroke was linked to decisions about referral onto a residential care rather than an inpatient rehabilitation pathway.

Similarly to the observed improvement, five studies found that clinician’s predictions about improvement (or rehabilitation potential) were an important and sometimes overriding factor in decision-making. Predicting discharge destination determined clinical priority and the care patients would receive; patients for discharge into residential care would become low priority for rehabilitation.^18,20^

### Organizational factors

Organizational factors, such as service acceptance criteria and workforce capacity, were found to influence decisions about stroke rehabilitation. Service pressures were discussed in seven studies from four different countries using a mix of methods (see Supplementary Table 1). This predominantly related to bed shortages; some participants described having to discharge patients before they felt they reached the end of inpatient rehabilitation,^16^ and limited bed availability was identified as a barrier to referring patients for postacute rehabilitation.^18,24^ Participants in Lam Wai Shun et al.’s^17^ study described having limited time to assess patients, creating pressure for quick decision-making based on a single encounter with patients. Similarly, participants in Longley et al.’s^10^ study described the challenges of working with people with cognitive impairments within time-limited services, when they may require longer to progress with rehabilitation. Johnson et al.^16^ observed multidisciplinary team meetings and found that a barrier to decision-making about discharge destination was lack of time for some members of staff to actually attend meetings. Staffing shortages were also found to be barrier for admitting patients for rehabilitation^23^ and restricted the amount of time clinicians were able to spend with each patient.^18^

In a study from the United States,^24^ insurance was found to be the biggest barrier in referring patients to the appropriate level of postacute care, thus affecting the decision of whether patients would receive ongoing rehabilitation. Insurance was also a factor in decisions to admit to some European stroke rehabilitation units;^25^ however, these findings are not found in countries with universal healthcare such as the United Kingdom. Proximity was a factor affecting decisions to refer to specific units; patients were more likely to be admitted to rehabilitation units in the same hospital as the acute unit in three sites in Putman et al.’s^25^ study. Clinicians were also aware that proximity to family was a factor influencing choice of rehabilitation unit.^23,24^

### Characteristics of individual clinicians

Awareness of clinicians’ own professional clinical discipline was cited as a factor that helped focus the evaluation of rehabilitation potential and sometimes was used to advocate for a patient to receive rehabilitation.^10,17^ Discharge planners found non-physician clinicians to be more influential than physicians when referring for rehabilitation in one study,^24^ indicating that professional discipline may have an important role; however, no detail is given about the specific roles of non-physicians and therefore it is unclear what type of expertise is preferred in this setting.^24^

Occupational therapists in Lam Wai Shun et al.’s^17^ study described how their clinical experience was a factor that influenced their decision-making. Assessment of rehabilitation potential and recovery was made by drawing on experiential knowledge. Experience, or lack thereof, was cited as a factor that challenged decisions regarding rehabilitation and participants expressed that additional skills were required when working in acute stroke.^10,18^

Clinician’s knowledge and awareness influenced decisions. Lam Wai Shun et al.^17^ found that clinicians referred to scientific evidence and clinical guidelines to aid decisions (although did not detail the guidance specifically); however, Lynch et al.^19^ found that lack of knowledge was a barrier for participants to refer patients to rehabilitation. They highlighted a belief from clinicians that rehabilitation was not suitable for patients with severe stroke, despite education sessions being provided demonstrating otherwise. Lack of knowledge about comorbid conditions (specifically dementia) was found to influence decisions about ongoing stroke rehabilitation for patients in Longley et al.’s^10^ study, with participants highlighting a lack of availability of extra training. Clinicians’ awareness of rehabilitation services also influenced which services patients were referred to and when.^17,18^ Johnson et al.^16^ identified that clinicians’ lack of knowledge about rehabilitation services available in the community resulted in delays in discharge, or patients not being referred for rehabilitation at all. In addition, fear of damaging relationships with rehabilitation providers prevented some clinicians from referring patients when they considered them unlikely to be accepted.^19^

Finally, five qualitative studies identified an emotional element to decision-making for clinicians, with participants wanting to give all patients a chance with rehabilitation while not challenging limited resources (see Supplemental Table 1). Participants in Longley et al.’s^10^ study described an element of “gut instinct” informed decision-making, particularly for less experienced clinicians. Luker et al.^18^ identified an “ethical strain” when attempting to provide equal levels of care; participants stated that they were aware that certain demographics of patients (e.g. severe stroke, older age) had more difficulty in acquiring postacute rehabilitation and yet also acknowledged that they provided more rehabilitation to younger patients.^18^

## Discussion

This systematic review of clinical decision-making about access to stroke rehabilitation found that a combination of patient and organizational factors and the characteristics of the decision-makers can influence decisions. It appeared that the most important patient-related factors were patients’ pre- and poststroke functioning (particularly whether they have prestroke dementia) and level of social support. Service pressures and clinicians’ own knowledge also influenced whether patients would be referred or admitted for rehabilitation. Surprisingly, five studies described an emotional element to decision-making, which highlights the challenge faced by clinicians when formal guidance is lacking. This review reveals the complexity of decision-making and the delicate balance of factors that may lead to a patient receiving, or not receiving poststroke rehabilitation.

The limitations of this review require consideration due to the mix of included studies. The low-quality appraisal scores of some included quantitative studies reflect a need for clearer reporting and more representative samples of participants in this area, for example, researchers could invite all people involved in discharge planning to participate from sampled services. Caution should be used when comparing their results to the highly appraised studies. There was heterogeneity in the organization of rehabilitation services and referral systems across the included studies which may limit the applicability of results, for example, some relied on external assessors selecting patients rather than patients being referred; some studies were carried out in generic acute or rehabilitation settings rather than stroke specific, and insurance was an influential factor in studies from countries requiring insurance to access healthcare. Organization of stroke services influences clinicians’ consideration about when to refer/admit patients for rehabilitation; service organization affects patient outcomes,^2,27^ and therefore consideration needs to be made when applying these results across services.

This review did find similarities across all nine countries covered by the included studies, which increases our confidence in the generalizability of our findings. The patient-related factors identified in this review are similar to those identified in a systematic review of patient-level studies that looked at prognostic factors influencing selection for rehabilitation,^8^ which supports our findings. This review builds on the existing literature by summarizing research from clinicians’ perspectives and addressing the organizational and individual clinician-level factors as well.

The review itself has a number of limitations. For one, search results were limited to English language. In addition, the search terms may have resulted in records being overlooked; the terms decision-making, clinical reasoning or clinical judgement were used based on previous studies and suggested search terms in databases, but despite this alternatives may still be used by some authors. There may have been ambiguity about what studies to include given the nature of the topic and the inclusion criteria, although all abstracts were reviewed by a researcher independent to the team in order to minimize this. One of the included studies was written by the authors of this review, which introduces elements of bias to the quality rating and importance given to certain factors. Again, quality was rated by an independent reviewer in order to minimize this bias.

Seven studies in this review were limited to single disciplines. Two were directly related to the experience of occupational therapists,^15,17^ with the others being focussed on the perspective of rehabilitation assessors or discharge planners, that is, the clinician deciding whether to refer or accept patients for rehabilitation. These roles are reflective of different healthcare systems and therefore not necessarily generalizable to all countries, for example, the United Kingdom. Evidence suggests that decisions to accept patients to rehabilitation (including patients with stroke) are variable across clinical disciplines,^28^ which indicates a need for multidisciplinary guidance about rehabilitation potential. Stroke rehabilitation is multidisciplinary,^1^ and therefore the decision of whether a patient receives rehabilitation should be informed by all perspectives; future research needs to reflect this.

An important finding was that no service reported using formal criteria to aid decisions for rehabilitation. In fact, Lynch et al.^19^ specifically explored whether a nationally recommended assessment tool^29^ was being used in practice to guide assessment and referral for rehabilitation. They determined that only one out of eight sites studied used the recommended tool as the criteria to determine rehabilitation requirements, and four sites did not consistently use any type of assessment criteria. They recommended that more interdisciplinary guidance is required in order to ensure patients receive equal access to stroke rehabilitation.^19^ Our findings reveal that this subjective approach to rehabilitation decision-making occurs across services and countries, and more comprehensive methods of supporting decision-making are required.

Information about inconsistencies in access to rehabilitation has implications for clinical practice. Evidence suggests that older stroke patients are less likely to receive evidence-based stroke care processes than younger patients,^30^ and this review identified age as a barrier for acceptance into rehabilitation services.^16^ Similarly, prestroke dementia has been associated with poorer outcomes;^7^ however, it is unknown whether this is due to lack of opportunities for rehabilitation. While there is some recent evidence suggesting that prestroke dementia influences clinical decisions for stroke rehabilitation,^10^ this review has identified the need to further explore this in order to close gaps in inequality of access. There is no evidence to restrict access to stroke rehabilitation for certain patients,^2^ and therefore there is a need to challenge these barriers to stroke rehabilitation.

This review highlights other barriers around access to stroke rehabilitation, particularly regarding clinicians’ own knowledge. Clinical decision-makers need to be aware that their perspective of patient-level and organizational factors, as well as their own individual characteristics, influences their decisions about stroke rehabilitation. Some of these barriers to rehabilitation are potentially modifiable by addressing staff knowledge deficits and attitudes to rehabilitation potential. Further studies on this topic require consideration of researcher influence, more representative samples of the study population and more specificity as to how factors positively or negatively influence decisions.

**Clinical messages**

- Decisions about referring/accepting patients into stroke rehabilitation are influenced by not only patient factors, but also organizational factors and characteristics of the clinician.
- Clinical decisions appear to take a subjective approach due to lack of clinical guidance about which patients should receive stroke rehabilitation.

## Supplemental Material

Supplemental_Material – Supplemental material for What factors affect clinical decision-making about access to stroke rehabilitation? A systematic review Click here for additional data file. Supplemental material, Supplemental_Material for What factors affect clinical decision-making about access to stroke rehabilitation? A systematic review by Verity Longley, Sarah Peters, Caroline Swarbrick and Audrey Bowen in Clinical Rehabilitation
