# Supplementary material for: What factors affect clinical decision-making about access to stroke rehabilitation? A systematic review
Source: Clin Rehabil. 2018 Oct 29;33(2):304–16. doi: 10.1177/0269215518808000 (PMC6348456; doi:10.1177/0269215518808000)
Supplement: Supplemental_Material – Supplemental material for What factors affect clinical decision-making about access to stroke rehabilitation? A systematic review [file Supplemental_Material.pdf]

Supplementary Table 1. Summary of factors influencing decisions

	Patient related factors								Organisational factors		Characteristics of individual clinicians			
Studies	Age	Pre- and post-stroke function	Type/ severity of stroke	Presence of dementia	Social/family support	Motivation	Demonstration of progress	Predictions about recovery / discharge	Service pressure	Insurance	Clinical discipline	Experience	Knowledge	Emotions
Burton et al. (2015)[21]			✓	✓		✓			✓					✓
Daniëls et al. (2002)[19]		✓				✓	✓							✓
Johnson et al. (2015)[23]	✓				✓				✓				✓	
Lam Wai Shun et al. (2017)[22]	✓	✓				✓	✓	✓	✓		✓	✓	✓	✓
Longley et al. (2018)[10]		✓		✓			✓	✓	✓		✓	✓	✓	✓
Luker et al. (2014)[14]	✓	✓	✓		✓	✓	✓	✓	✓			✓	✓	✓
Lynch et al. (2017)[16]				✓	✓		✓	✓						

Lynch et al. (2016)[15]			✓	✓									✓	
Hakkennes et al. (2013)[17]		✓		✓	✓									
Hasenbein et al. (2002)[31]	✓										✓			
Kennedy et al. (2012)[18]	✓							✓	✓					
Magdon-Ismail et al. (2016)[24]					✓				✓	✓	✓			
Putman et al. (2007)[20]		✓		✓	✓	✓				✓				

Appendix 1

MEDLINE Search strategy

Search Order	Search terms incorporating Boolean terminology	Article yield
S1	(MH "Stroke") OR (MH ”cerebrovascular accident) OR (MH ”CVA”)	237,055
S2	(MH “rehabilitation”)	307,070
S3	(MH “therapy”)	3,702,752
S4	S2 OR S3	3,909,165
S5	(MH “Decision making”)	163,768
S6	(MH “clinical reasoning”)	2,598
S7	S5 OR S6	165,750
S8	S1 AND S4 AND S7	1,293
